# On the effect of electronic patient portal on primary care utilization and appointment adherence

**DOI:** 10.1186/s12911-018-0669-8

**Published:** 2018-10-16

**Authors:** Xiang Zhong, Muxuan Liang, Reynerio Sanchez, Menggang Yu, Pamela R. Budd, Julie L. Sprague, Marvin A. Dewar

**Affiliations:** 1 Department of Industrial and Systems Engineering, University of Florida, 482 Weil Hall, PO BOX 116595, Gainesville, FL 32611-6595 USA; 2 Department of Statistics, University of Wisconsin – Madison, Madison, WI USA; 3 Department of Biostatistics & Medical Informatics, University of Wisconsin – Madison, Madison, WI USA; 4 UF Health, Gainesville, FL USA

**Keywords:** Patient portal, Primary care utilization, Appointment adherence, Disease process, Panel-DID, Causal inference

## Abstract

**Background:**

The objective of this study was to investigate the impact of patient portal adoption on patients’ primary care utilization and appointment adherence.

**Methods:**

We conducted a retrospective observational study using a panel difference-in-differences (DID) framework to investigate the use of primary care services by patients, adjusting for their disease burden and allowing for time-dependent portal effect. A large dataset with 46,544 patients of University of Florida (UF) Health during the study period July 2013 – June 2016 was used. The main outcome measures are disease burden adjusted rates of office visits arrived, no-show, and cancellation to primary care physicians (PCPs) per quarter between patient portal adopters (denoted as users) and non-users.

**Results:**

At the time of adoption, the quarterly PCP office visit rate ratio (RR) of patient portal users to non-users was 1.33 (95% CI, 1.27–1.39; *p* < 0.001). The RRs were between 0.94 to 0.99 up to four quarters after portal adoption (*p* = 0.749, 0.100, 0.131, and 0.091, respectively), and were significantly less than one at the seventh (RR =0.82; 95% CI, 0.73–0.91; *p* < 0.001) and the eighth (RR = 0.80; 95% CI, 0.70–0.90; *p* < 0.001) quarters post adoption. The quarterly no-show rates of the users were significantly smaller (RRs were between 0.60 and 0.83) except for the seventh, eighth and tenth quarters post adoption. In these three quarters, the no-show rates were not significantly changed (*p* = 0.645, 0.295, and 0.436, respectively). Quarterly cancellation rates were not significantly affected by portal adoption (*p* > 0.05 for all cases).

**Conclusions:**

Patient portal users’ disease burden adjusted PCP office visit rate was significantly reduced in one and a half year and thereafter post portal adoption. PCP appointment no-show rate was also significantly reduced and cancellation rate was not affected, implying improved care engagement of patients.

## Background

A spate of studies have demonstrated that patients who are more actively engaged in their own health care experience better health outcomes and incur lower costs [1]. As a result, a growing number of public and private health care organizations are employing strategies to improve patient engagement. Among them, patient portals are recognized as a promising mechanism to support greater patient engagement by increasing communication between patients and providers, and enabling patients to make competent and well-informed decisions. Empowered by the rapid development of health information technology and facilitated by the US federal government (e.g., the Health Information Technology for Economic and Clinical Health Act, which authorized incentive payments to physicians who demonstrated “meaningful use” of health information systems [2]), patient portals are now widely available and increasingly being adopted by patients and providers.

Patient engagement is generally referred to as a broad concept that combines a patient’s knowledge, skills, ability and willingness to manage their own health and care with interventions designed to promote positive patient behaviors [1]. A state-of-the-art review of patient portals and patient engagement including topical areas of patient adoption, provider endorsement, health literacy, usability, and utility was presented in [3]. In particular, patients with access to their personal health records are hypothesized to become more educated consumers of care and better informed to engage in self-management, thereby reducing health care utilization ultimately [4–6]. However, research findings drew inconsistent conclusions regarding the impact of portal usage on patients’ care utilization. For instance, it was found in [7] that increased outpatient utilization was associated with patient portal registration. Portal registration and usages of online messaging and record viewing were identified being associated with more pediatric ambulatory visits [8], and the release of test results may produce unnecessary anxiety and increase the rate of patient visits [9]. Conversely, another set of studies did not find any association between patient portal activities (e.g., secure messaging) and number of primary care office visits [10–13], or suggested that access to patient portal can enhance care delivery efficiency and even substitute for some face -to -face health care services [14–19].

The majority of the above-mentioned studies involved a small number of patients or were designed to collect observations from a relatively short time period. It is worth noting that patients were more likely to receive information about registering for portal during or after a clinic visit, and some increases in care utilization may be attributed to resolving the triggering health care condition, which gives rise to the health care encounter at which the portal registration occurred. Such facts need to be factored into the investigation of the portal effect. We are not aware of any extant study that analyzed this natural impact of presenting disease, which may confound any subsequent evaluation of the portal effect. Additionally, patients’ health conditions could be a confounding factor that affects a patient’s choice of becoming a portal user and consequently asserts a positive influence on patients’ care utilization. Patients who bear heavy disease burden, indexed by more active problems recorded in the electronic medical record (EMR), exhibited a significantly higher level of ambulatory care utilization. To accurately gauge the association between *patient portal adoption* and any impact on the subsequent *use of clinical services*, it would be instructive to track a large, longitudinal panel of patients across multiple clinics to assess the independent impact of health conditions and patient portal adoption on health care service utilization.

In addition to primary care office visit rates, in this study, we consider office visit appointment no-show as an indicator of patient engagement. Missed appointment behavior has been chosen to serve as an indicator for patient adherence behaviors in several literatures [20–24]. Specifically, adherence to scheduled clinic visits was used as an objective proxy for adherence to medication between clinic visits [23, 24], and repeatedly missing appointments has been shown to lead to non-adherence to medication, faster disease progression, and treatment failure [22]. Patient portals enable patients and providers to share timely and pertinent information, and the messaging function of portal is also used as a communication tool to deliver reminders for preventive care and appointments [3]. A few works investigated whether portal enrollment is significantly related to decreases in rates of appointment no-shows. In [25], it was reported that monthly no-show rates across seven Duke Medicine clinics were significantly reduced among patients who registered for portal use, suggesting that in combination with an email reminder feature, portal technology may have an important and beneficial effect on clinic operations. Similar promising results were also found in [26–30]. However, the studies presented therein cannot establish a causal relationship between portal adoption and no-show reduction. Therefore, in our work, we used longitudinal data generated by a large patient cohort, and proposed a causal inference framework, aiming to test the hypotheses that 1) patients who adopt patient portal would decrease their disease burden adjusted use of primary care office visits, and 2) adopting portal improves PCP appointment adherence, which implies better care engagement of patients.

## Methods

### Study setting

The study was conducted at UF Health, a medical network associated with the University of Florida (UF). The UF Health network includes two academic hospitals and several other hospitals and facilities in North Central Florida. In 2011, UF Health started offering “MyUFHealth,” also known as MyChart® by Epic®. MyUFHealth is an electronic patient portal that provides patients a secure and convenient way to access portions of their medical records (e.g., released test results, after visit summary), communication with the clinical service providers using secure messaging, request prescription refills, and management of outpatient appointments. MyUFHealth is available to patients who are seen in the UF Health network at Gainesville or Jacksonville hospitals and physician outpatient practices. MyUFHealth pediatric proxy for children under 18 years old is also available and can be established in the UF Health Physicians clinics. Proxy access allows a parent (or guardian) to log into their personal MyUFHealth account, and then connect to the MyUFHealth account of their child. Therefore, children under 18 years old can also be portal users in this study.

**Fig. 1:**
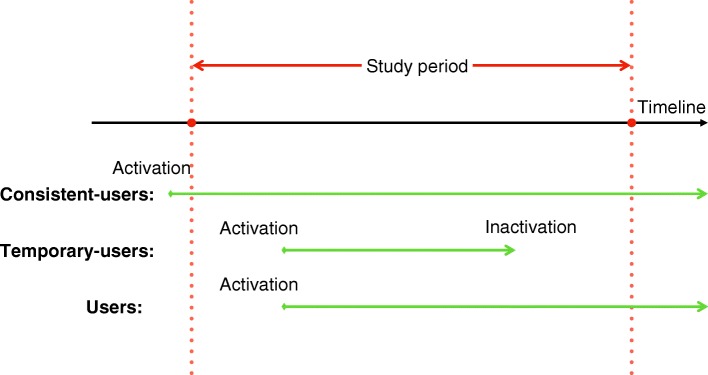
Illustration of consistent-users, temporary-users, and users

The data protocol used for the retrospective observational study was approved by the UF Institutional Review Board. The study period was from July 1st, 2013 to June 30th, 2016, i.e., fiscal years 2013–2015. We denote January – March as Q1, April – June as Q2, July – September as Q3, and October – December as Q4. For instance, Y13Q3 stands for the third quarter of the year 2013. The study population consisted of 46,544 UF Health patients who had at least one visit to UF Health Physicians clinics during the study period. Among these 46,544 patients, more than 95% of them came from North Central Florida (based on zip-code categorization), and more than 70% of them were from Alachua County, where UF Health is the major care provider. To classify patients, we only denote patients who adopted portal during the course of the study, and kept the active status until the end of the study as users, which were 9049 patients out of the whole population. Five thousand four hundred twelve patients adopted portal before the study period, and were nominated as consistent-users. Additionally, there were 715 temporary-users, who became inactive by the end of the study. Meanwhile, 31,368 patients never registered for patient portal and were denoted as non-users. The patient classification can be found in Fig. 1.

We further define *new patients* as who have not received any professional services, i.e., Evaluation and Management (E/M) service or other face-to-face service (e.g., surgical procedure) from the physician or physician group practice (family medicine or primary care) within the previous 3 years in the UF Health network. This definition is in consistent with the Current Procedural Terminology (CPT) [31]. Because patients were more likely to receive the portal activation code during or after an office visit, there was a resulting mechanical increase in the office visit rate for portal users at the quarter of adoption and its surrounding quarters (trends similar to the figure presented in [7] were witnessed). To eliminate the nested effect, we limited our focus to new patients, for whom we can identify the time they recorded a new diagnosis, allowing some assessment of the natural disease process. We used the visit type categorization “New” in EMR to pin down patients’ first-time UF Health encounters. Notably, patients typically adopted portal at the time of their new visits or right after their new visits. To ensure a before-after portal adoption contrast, we only focused on new patients who had their new visits and portal adoption (users only) after Y13Q3. We also excluded patients who were consistent-users and temporary-users. This inclusion criteria led to 15,659 non-users and 5494 users identified.

We were able to obtain information from the EMR and administrative databases on patients’ age, gender, race/ethnicity, marital status, insurance status, and ambulatory care utilization (telephone encounters, and office visits to PCP and specialty care clinics within the care network). We also recorded patients’ active problem number (APN). The active problem list in EMR records a patient’s major health conditions, including both chronic conditions and ongoing conditions that are resolvable but are important for physicians’ to make clinical decisions. The active problem list is typically reviewed and updated if needed at each patient encounter, and is used as a proxy for an individual’s disease burden in this study. Monthly portal access events and clinical service usage of individual patient were generally not frequent, so data were consolidated by quarter. Admittedly, the data set only includes patient ambulatory care utilization within the UF Health network. However, UF Health is the leading care provider in the region under investigation. Additionally, more than 95% of patients in this study were insured. The insurers typically request patients to select their primary care physician (e.g., Blue Cross Blue Shield, Commercial and Managed Care), or seek care with participating providers (e.g., Medicare and Medicaid). These suggest that primary care services rendered to patients outside of the UF Health network would be very limited. Therefore, the primary care utilization within UF Health is used as an indicator of the overall primary care utilization of patients.

### Propensity score matching

As an observational study, the initial patient characteristics of users and non-users were dissimilar, and we first calculated propensity scores by estimating the probability of becoming a user using logistic regression. Confounders that potentially affect both outcomes (primary care service utilization) and exposure to the intervention (becoming a portal user) include time-invariant variables (age, gender, race, marital status, insurance status), and time-varying variables (APN and PCP office visit arrived, no-show, and cancellation).

**Fig. 2:**
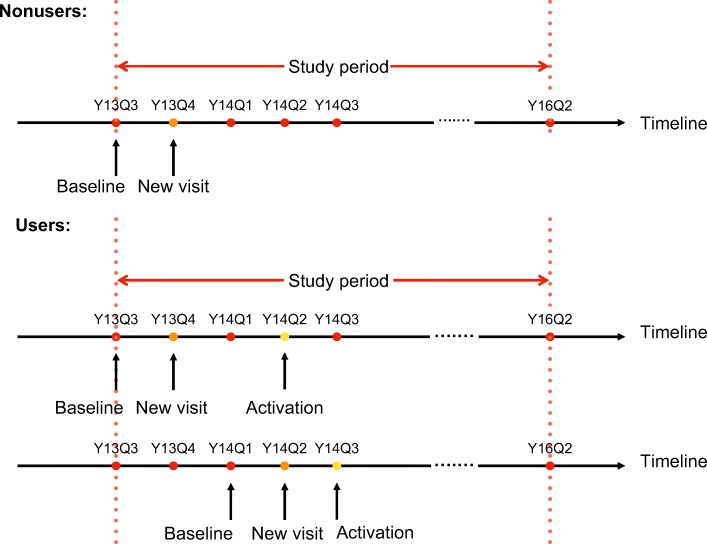
Illustration of baseline for matching non-users to users

As an observational study, both events -- becoming a new patient of UF Health and adopting patient portal -- happened throughout the study period, rather than at a fixed time. A challenge for matching emerges: if matched at the beginning of the study period, individual patients’ time-varying characteristics could diverge dramatically before the treatment started. Therefore, a baseline for each patient is defined for this study as the time (quarter) right before adopting portal, or before having the new visit, whichever was earlier. For instance, if a patient had his/her new visit in Y13Q4, and adopted portal in Y14Q1, then, the baseline is chosen as Y13Q3. The baseline definition is illustrated in Fig. 2.

To match patients, the baseline APN was discretized into three categories, characterized by `0′, `1–4′, and `5 and more’ active medical problems. Summation of PCP office visits (including new and other office visit types, if any) arrived at baseline were categorized as `0′, `1′, and `2 and more’, and that of no-show, and cancellation were dichotomized into `0′, and `one and more’. Discretization helps eliminate outliers and adjust the variance structure of count data.

**Fig. 3:**
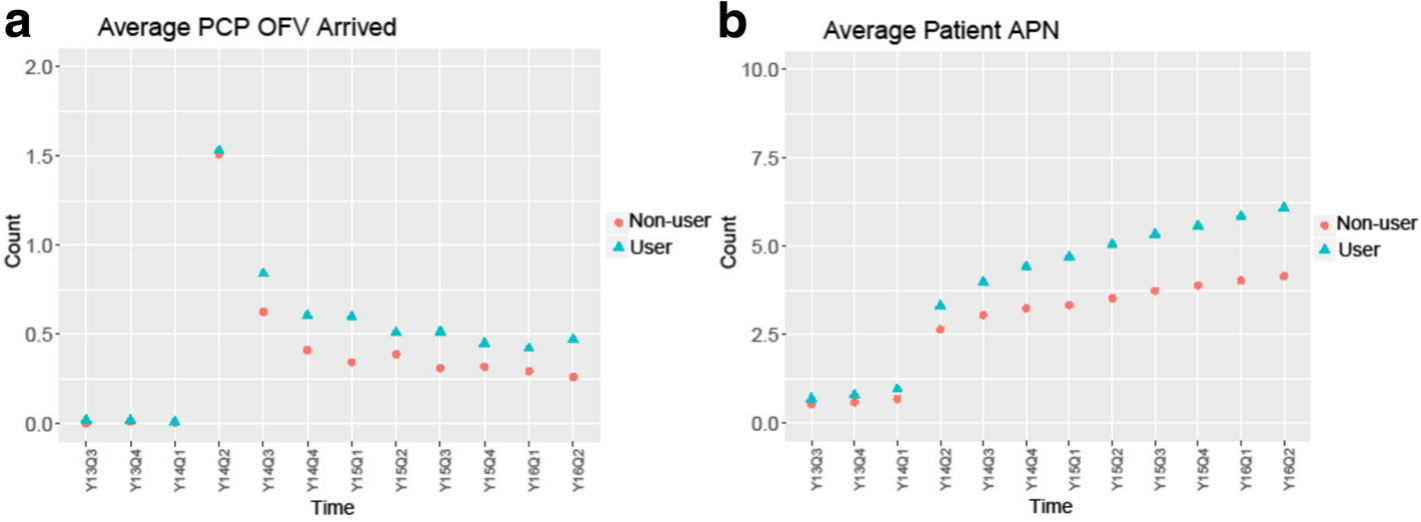
Comparison of portal users and matched non-users. **a** Average number of primary care clinic office visit (OFV) arrived in each quarter. **b** Average active problem number in each quarter

The matching was performed using propensity scores with the nearest neighbor selection criteria. To ensure the quality of matching, descriptive statistics of the confounding variables were calculated and tested using Chi-square tests (see Table 1). In Fig. 3, we illustrate a comparison of a subpopulation of portal users and matched non-users. The subpopulation included users who had their new visits during Y14Q2 and their portal adoption during Y14Q2 - Y14Q3, and non-users who had their new visits during Y14Q2 with characteristics matched with users at baseline Y14Q1. Notably, patients typically received the portal activation code during or after an office visit, and the office visit rate at the quarter of enrollment and its surrounding quarters were significantly higher than average due to the nature of disease process. Additionally, although matched at the baseline, users’ and non-users’ active problem numbers can diverge significantly. To elicit the effect of patient portal, the time-dynamic feature of disease burden needs to be captured, which motivates a causal inference study accounting for such time-varying confounders.

**Table 1:** Characteristics of the unmatched and matched portal non-users and portal users

	Non-users		Users			Matched Non-users		
	*n* = 15,659		*n* = 5494		*p*-value	*n* = 5494		*p*-value
Time-Invariant Variables								
Age Categories					< 0.05			0.78
0–18	1944	12.4%	57	1.0%		58	1.1%	
19–30	4328	27.6%	1619	29.5%		1619	29.5%	
31–45	3764	24.0%	1781	32.4%		1727	31.4%	
46–64	3755	24.0%	1478	26.9%		1528	27.8%	
65+	1868	11.9%	559	10.2%		566	10.3%	
Sex					< 0.05			0.70
Female	8703	55.6%	3636	66.2%		3656	66.6%	
Male	6956	44.4%	1858	33.8%		1838	33.4%	
Race					< 0.05			0.95
Asian	502	3.2%	249	4.5%		233	4.2%	
Black	4146	26.4%	735	13.4%		716	13.0%	
Hispanic	70	0.4%	21	0.4%		19	0.3%	
Other	945	6.0%	394	7.2%		387	7.0%	
Unknown	795	5.1%	191	3.5%		191	3.5%	
White	9201	58.8%	3904	71.1%		3948	71.9%	
Marital Status					< 0.05			0.48
Divorced/Separated	757	4.8%	257	4.7%		254	4.6%	
Married	4743	30.2%	2440	44.4%		2437	44.4%	
Other	151	1.0%	36	0.7%		25	0.5%	
Partner	78	0.5%	39	0.7%		28	0.5%	
Single	8205	52.5%	2288	41.6%		2311	42.1%	
Unknown	1356	8.6%	350	6.4%		367	6.7%	
Widowed	369	2.4%	84	1.5%		72	1.3%	
Time-Varying Variables								
Insurance Type Baseline					< 0.05			0.87
Blue Cross Blue Shield	6172	39.4%	3125	56.9%		3152	57.4%	
Commercial/Managed Care	2899	18.5%	1038	18.9%		1024	18.6%	
Federal/Military	88	0.6%	28	0.5%		29	0.5%	
Medicaid	3561	22.7%	558	10.2%		551	10.0%	
Medicare	2300	14.7%	551	10.0%		564	10.3%	
No Record	36	0.2%	24	0.4%		23	0.4%	
Other Program	18	0.1%	4	0.1%		0	0.0%	
Self-pay	580	3.7%	160	2.9%		150	2.7%	
Worker’s Compensation	5	0.0%	6	0.1%		1	0.0%	
Active Problem Number Baseline					< 0.05			0.83
“0”	6169	39.4%	1993	36.3%		1999	36.4%	
“1–4”	6971	44.5%	2537	46.2%		2555	46.5%	
“5+”	2519	16.1%	964	17.5%		940	17.1%	
PCP Office Visit Arrived Baseline (per Quarter)					0.21			0.19
“0”	15,490	99.2%	5449	99.3%		5452	99.2%	
“1”	71	0.5%	24	0.4%		20	0.4%	
“2+”	59	0.4%	12	0.2%		22	0.4%	
PCP Office Visit No-show Baseline (per Quarter)					< 0.05			0.43
“0”	15,524	99.1%	5469	99.5%		5462	99.4%	
“1+”	135	0.9%	25	0.5%		32	0.6%	
PCP Office Visit Canceled Baseline (per Quarter)					< 0.05			0.06
“0”	15,379	98.2%	5420	98.7%		5387	98.2%	
“1+”	280	1.8%	74	1.3%		97	1.8%

### Difference-in-differences (DID) models

In our study, we recorded both the time when patient *i* had their new PCP office visit (time of clinic enrollment: *NewIndex*_*i*_) and the treatment time (time of portal activation *AdoptIndex*_*i*_). *NewIndex*_*i*_ and *AdoptIndex*_*i*_ for each patient *i* can be different. Both disease effect and treatment effect are time varying, and we are interested in the effect of treatment over time. Thus, we proposed a model using a panel-DID framework accounting for heterogeneous enrollment and treatment times. All the DID analyses in this study were conducted using users and matched non-users.

To address the confounding effects, discretized active problem number is incorporated as a time-varying covariate *APN*_*it*_. Other confounders are represented by *X*_*i*_, an ensemble of the time-invariant control covariates (age, gender, race, marital status, and insurance status) for the *i*^*th*^ patient. To accommodate the special variance structure of count responses, a generalized linear model with *Poisson distribution adjusted for over-dispersion* is proposed:

1 $$ g\left(E\left[{Y}_{i t}\right]\right)={\gamma User}_i+{\sum}_{\tau =0}^T{\lambda}_{\tau } Tim{e}_{i\tau (t)}+{\sum}_{k=0}^{T-1}{\theta}_k{D}_{i k(t)}+{\sum}_{k=0}^{T-1}{\beta}_k{P}_{i k(t)}+\alpha {APN}_{i t}+\eta {X}_i. $$

In model (1), *g*(·) is a log-link function, *Y*_*it*_ is the outcome variable (number of office visits arrived for the *i*^*th*^ patient at time *t*), and *E*[*Y*_*it*_] represents the expectation of the corresponding outcome. *User*_*i*_ is a dummy variable to indicate whether the *i*^*th*^ patient belongs to the treatment group or not. Let index *t* denote the time the measurements (responses and covariates) were recorded, *t* = 0, 1, …, *T*. We let *t* = 0 represent the beginning of study period (Y13Q3), and *T* = 11 represent Y16Q2. Let *Time*_*iτ*(*t*)_ be a dummy variable to adjust for fixed effect of time *λ*_*τ*_, and *Time*_*iτ*(*t*)_ = 1 if and only if *τ* = *t*, *t* = 0, …, *T*.

**Fig. 4:**
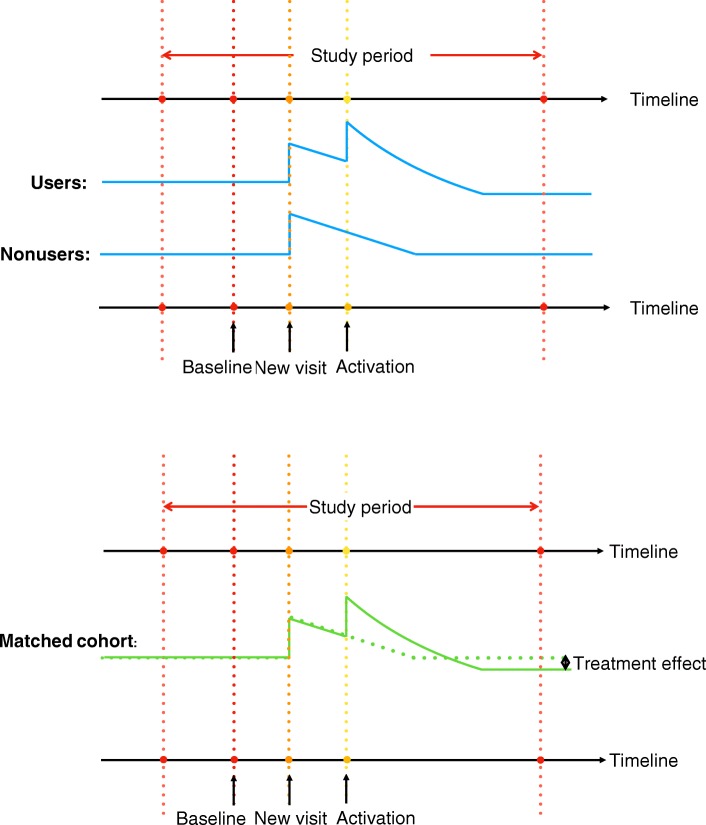
Illustration of the panel-DID model design and the treatment effect

Patients could have their new PCP office visits during any *t* such that *T* ≥ *t* > 0. To incorporate the disease process which affects both users and non-users, a dummy variable *D*_*ik*(*t*)_ is introduced to represent if the absolute time *t* is *k* quarters post becoming new patients, and *D*_*ik*(*t*)_ = 1 if and only if *t* − *NewIndex*_*i*_ = *k*. The coefficient *θ*_*k*_ is the disease effect at time lag-k, *k* = 0, …, *T* − 1. To investigate the role time-lag plays in treatment (portal adoption) effects, a dummy variable *P*_*ik*(*t*)_ is introduced, and *P*_*ik*(*t*)_ = 1 if and only if *t* − *AdoptIndex*_*i*_ = *k* and *User*_*i*_ = 1. Consequently, *β*_*k*_ is the lag-k treatment effect, i.e., the difference-in-differences between the users and non-users at the *k*^*th*^ quarter post portal adoption. Specifically, *β*_0_ is the treatment effect at the time of adoption. Figure 4 is presented to illustrate the design of the DID analysis.

### No-show and cancellation comparisons

Using the proposed model, the time-varying portal effect on care utilization can be analyzed. In addition, we evaluated the prevalence of appointment no-shows and cancellations between the user and non-user groups. To this end, *Y*_*it*_ is replaced by the count of PCP appointment no-shows for patient *i* at quarter *t* and we fitted Model (1). With this modification, another model was run with the same structure but the response *Z*_*it*_ is the count of PCP office visit appointments made that were not no-show.

2 $$ g\left(E\left[{Z}_{i t}\right]\right)={rUser}_i+{\sum}_{\tau =1}^T{l}_{\tau } Tim{e}_{i\tau (t)}+{\sum}_{k=0}^{T-1}{c}_k{D}_{i k(t)}+{\sum}_{k=0}^{T-1}{b}_k{P}_{i k(t)}+{aAPN}_{i t}+{\omega X}_i. $$

No-show rate is defined as the number of appointment no-shows divided by the number of appointments scheduled for a given duration. At patient-level, we denote the number of appointment no-shows for patient *i* at the *k*^*th*^ quarter post adoption as *Y*_*ik*(*t*)_, and denote the number of appointments made that were not no-show for as *Z*_*ik*(*t*)_. *P*_*ik*(*t*)_ denotes whether time *t* is the *k*^*th*^ quarter post adoption or not, and *X*_*i*_ reflects a patient’s individual time-invariant attributes. For users at the *k*^*th*^ quarter post intervention, we are interested in the patient-level no-show rate denoted as

3 $$ {r}_{ik(t)}=\frac{E\Big[{Y}_{ik(t)}\left|{P}_{ik(t)}=1,{X}_i\right]}{E\left[{Y}_{ik(t)}\left|{P}_{ik(t)}=1,{X}_i\right]+E\right[{Z}_{ik(t)}\left|{P}_{ik(t)}=1,{X}_i\right]}. $$

For brevity purposes, we omit the quarter index *k* and introduce ${r}_i^1$ and ${r}_i^0$ to represent a user’s no-show rate and a counterfactual case (if that patient is a non-user):

4 $$ {r}_i^1=\frac{E\Big[{Y}_i\left|{P}_i=1,{X}_i\right]}{E\left[{Y}_i\left|{P}_i=1,{X}_i\right]+E\right[{Z}_i\left|{P}_i=1,{X}_i\right]}, $$

5 $$ {r}_i^0=\frac{E\Big[{Y}_i\left|{P}_i=0,{X}_i\right]}{E\left[{Y}_i\left|{P}_i=0,{X}_i\right]+E\right[{Z}_i\left|{P}_i=0,{X}_i\right]}. $$

Then, the no-show rate ratio of patient *i* comparing before and after portal adoption is denoted as $R{R}_i=\frac{r_i^1}{r_i^0}.$ Before moving forward, we introduce another set of variables: ${\rho}_i^1=\frac{E\Big[{Y}_i\left|{P}_i=1,{X}_i\right]}{E\Big[{Z}_i\left|{P}_i=1,{X}_i\right]},$ ${\rho}_i^0=\frac{E\Big[{Y}_i\left|{P}_i=0,{X}_i\right]}{E\Big[{Z}_i\left|{P}_i=0,{X}_i\right]},$ and ${\Omega}_i=\frac{\rho_i^1}{\rho_i^0}.$

${\rho}_i^1$ and ${\rho}_i^0$ can be roughly interpreted as the average number of no-shows that will occur to successfully complete one appointment. We denote *θ* and *δ* as the coefficients that are independent of *P*_*i*_ and *X*_*i*_; then, based on models (1) and (2), ${\rho}_i^1={\exp}^{\theta -\delta +\left({\beta}_k-{b}_k\right)+\left(\eta -\omega \right){X}_i}$, ${\rho}_i^0={\exp}^{\theta -\delta +\left(\eta -\omega \right){X}_i}$, and ${\Omega}_i={\exp}^{\beta_k-{b}_k}$. It can be easily proved that *RR*_*i*_ < 1iff. Ω_*i*_ < 1. If the no-show rate is reduced after portal adoption, then Ω_*i*_ < 1, and vice versa. Therefore, testing the hypothesis H_0_: *RR*_*i*_ < 1 is equivalent to testing the hypothesis H_0_^’^: Ω_*i*_ < 1. Specifically, *β*_*k*_ is the difference-in-differences of PCP appointment no-shows and *b*_*k*_ is difference-in-differences of PCP appointments made that were not no-show between the users and non-users at the *k*^*th*^ quarter post adoption. If ${\exp}^{\beta_k-{b}_k}<1$, then, it can be concluded that accessing portal is associated with a reduced no-show rate. The differences of coefficients of treatment effect between the two models, i.e., *β*_*k*_ − *b*_*k*_, *k* = 0, …, *T* − 1, can be compared and formally tested. Similarly, *Y*_*ik*(*t*)_ can be the number of PCP appointment cancellations for patient *i* at quarter *k* post intervention to investigate the ratio of cancellation rate in the user group to that in the non-user group.

## Results

In this study, all statistical analyses were performed using R version 3.3.1 with 2-sided statistical tests at a .05 significance level. From fiscal year 2013 to 2015, the percentage of patients who adopted portal increased steadily from 17.5 to 31.4%, and the average of PCP office visit rates ranged from 0.50–0.65 quarterly. It was observed that albeit matched at the baseline, the active problem number at the end of the study differed significantly when comparing users versus non-users. Despite the general trend that the active problem number increased over time, the user group comprised 53.1% patients with more than four chronic problems (compared to 40.2% of the matched non-user group), and had more patients bearing ten or more chronic problems (18.2% vs. 12.2%). It echoes Fig. 3a and b that when unadjusted, portal users exhibited more office visits, and patients who adopted portal access maintained more active problems than did portal non-users.

### Patient portal adoption

The characteristics of the unmatched and matched cohorts are shown in Table 1. When employing propensity scoring to match non-users to portal users, we noticed that individuals who enrolled in patient portal were mostly middle aged (between 31 and 64 years) and more likely to be female. The proportion of teenage patients was greater in the non-user cohort, possibly due to impacts from the proxy issue. Moreover, patients whose marital status was recorded as “Married” were more likely to adopt patient portal. Medicare and Medicaid patients, and patients with race self-reported as Black or African American were less likely to be portal adopters. These observations are consistent with previous studies on social disparities in enrollment and use of patient portals [32–37]. Portal adoption was also associated with the baseline number of active medical problems on the problem list (*p* < 0.05).

### Patient care utilization and appointment adherence

Using the panel-DID models, we compared the utilization of PCP office visits between portal users and non-users. The RR of users to non-users attributable to treatment effect at the time of adoption was 1.33 (95% CI 1.27–1.39; *P* < 0.001). This boosting effect declined immediately after adoption (see Table 2). Specifically, the count of PCP office visits was similar between users and non-users in the first four quarters after portal adoption, and that of users was significantly lower than non-users in the seventh (RR =0.82; 95% CI, 0.73–0.91; *p* < 0.001) and the eighth (RR = 0.80; 95% CI, 0.70–0.90; *p* < 0.001) quarters post adoption. Insignificant differences for the ninth and tenth quarters post adoption, possibly due to insufficient sample size (only about 10% of users defined in the study adopted portal at the second quarter (Y13Q4) and had a history of ten quarters post adoption) were observed. The trend of RR attributable to lag-k treatment effect can be seen in Table 2. The observation is not consistent with those in [7] and other analyses which employed the same methodologies without accounting for the natural process of disease confounder.

**Table 2:** Quarterly rate ratio [Users]/[Non-users] of PCP office visit arrived, no-show and cancellation

Quarter Post Adoption	PCP Office Visit Arrived			PCP Office Visit [no-show]/[appointments made not no-show]			PCP Office Visit [canceled]/[appointments made not canceled]		
	Estimated	95% CI	*p*-value	Estimated	95% CI	*p*-value	Estimated	95% CI	*p*-value
0	1.330	(1.271, 1.389)	< 0.001	0.663	(0.567, 0.759)	< 0.001	0.953	(0.871, 1.034)	0.255
1	0.991	(0.935, 1.047)	0.749	0.827	(0.695, 0.959)	0.010	1.051	(0.944, 1.158)	0.351
2	0.950	(0.890, 1.010)	0.100	0.752	(0.612, 0.892)	< 0.001	1.053	(0.933, 1.172)	0.386
3	0.950	(0.885, 1.015)	0.131	0.806	(0.645, 0.962)	0.018	0.969	(0.848, 1.090)	0.616
4	0.941	(0.871, 1.010)	0.091	0.784	(0.612, 0.956)	0.014	0.915	(0.790, 1.040)	0.182
5	0.901	(0.828, 0.974)	0.008	0.685	(0.509, 0.862)	< 0.001	1.042	(0.890, 1.195)	0.586
6	0.920	(0.836, 1.003)	0.060	0.658	(0.466, 0.850)	< 0.001	0.908	(0.755, 1.060)	0.234
7	0.819	(0.734, 0.905)	< 0.001	0.932	(0.649, 1.214)	0.635	0.961	(0.777, 1.145)	0.678
8	0.804	(0.704, 0.903)	< 0.001	0.835	(0.525, 1.144)	0.295	1.062	(0.825, 1.298)	0.609
9	0.911	(0.773, 1.049)	0.209	0.603	(0.291, 0.915)	0.013	1.081	(0.795, 1.366)	0.580
10	0.905	(0.696, 1.113)	0.371	0.781	(0.232, 1.331)	0.436	1.074	(0.641, 1.508)	0.738

Additionally, we examined the relationship between portal usage and appointment adherence. In particular, we chose our proxies for PCP appointment no-show and cancellation rates as outcomes (see Table 2). The no-show rate proxies of the user group were significantly lower than that of non-users. RRs were between 0.60 and 0.83 for eight out of eleven quarters, and for the remaining three quarters, the differences were not significant (detailed *p*-values can be found in Table 2). The differences in cancellation rate proxies were not significant (with *p* > 0.05 for all cases). Overall, patient appointment adherence was improved after portal adoption.

### Sensitivity analysis

**Fig. 5:**
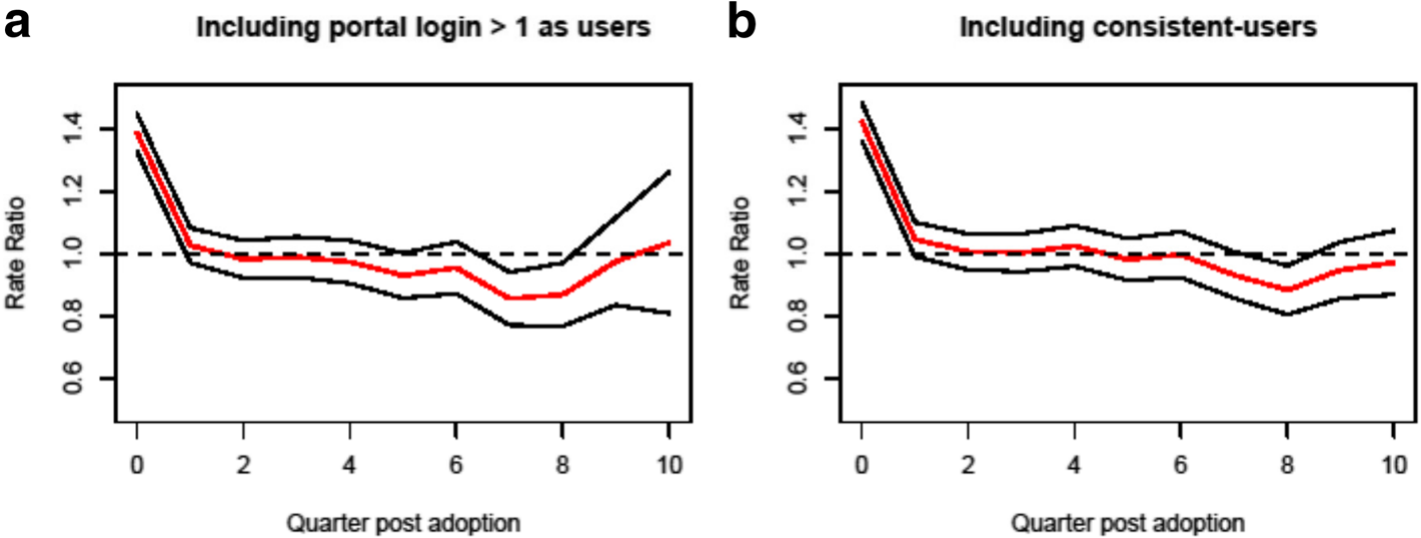
Quarterly rate ratio [Users]/[Non-user] of primary care clinic office visits arrived. **a** Including users who had more than one portal login. **b** Including users and consistent-users

Robustness is the degree to which a model performs effectively even if some of its variables are modified. First, we analyzed robustness using an alternative measure of portal usage, where we define users as new patients who exhibited at least two portal logins (one for initial activation and then a subsequent login) during Y13Q4 – Y16Q2. Those who did not have at least two logins were considered as non-users even if the patient qualifies the inclusion criteria for “user” defined previously. This modification guarantees that the alternative definition is close to the original one but with minor changes, and would eliminate the coding errors when patients’ portal status was mischaracterized as “Activated.” The corresponding results were similar to the original model with respect to RR estimates and significance, which confirmed the robustness of the underlying model. Furthermore, we adjusted the inclusion criteria by including new patients who were consistent-users (who adopted portal before or right at the beginning of the study period). We identified 744 patients and assumed for those consistent-users that their adoption happened at the first quarter (*AdoptIndex*_*i*_ = 0). Adding consistent-users to the user group produced minor discrepancies between the two sets of panel-DID models concerning the absolute values and significance of coefficient estimations, but the trends of treatment effects in both sets of models were consistent. The trends of RR attributable to lag-k treatment effect for PCP office visit arrived with different inclusion criteria, (a) users with more than one portal login, and (b) including users and consistent-users are exhibited in Fig. 5.

## Discussion

A possible driver of portal adoption is the anticipation of intensified care demands. Patients who find themselves in need of clinical services may intentionally sign up for portal access and thus gain more frequent access to care. This appears contradictory to the outcomes from the DID models, but is in fact not inconsistent, as the portal effect in the DID models has been adjusted by the time-varying covariate active problem number. Overall, the increase of care utilization in the user group might be due to more health problems identified and diagnosed.

On the impact of portal on patients’ care utilization, we concluded that patients with portal access had no significant change in use of in-person PCP visits immediately after their adoption, and exhibited reductions in care utilization adjusted by their disease burden in about 1 or 2 years later. Such conclusions differ from the results of prior studies [7, 8, 38, 39], all of which did not incorporate the natural process of disease and disease burden. For these studies, cohort matching was conducted at the baseline level. Therefore, such models cannot closely reflect the dynamics of the time-varying confounders such as disease burden, which has been proved to be critically associated with care utilization. One other caveat of previous analyses is their lack of differentiation between the portal adoption process (the workflow of portal activation) and the natural process of disease (diagnosing, resolving, and healing). In our study, we limited our focus to new patients, which enabled us to have a distinct separation of disease process and portal process. The panel-DID framework was capable of detecting the trend of portal effect which might not be time-homogeneous. In our robustness check, we deliberately included the experienced users who had relatively long exposures to both PCP office visits and portal access. The findings were practically invariant with different inclusion criteria and definitions of users.

Another advantage of our model is its generic framework and flexibility to test different hypotheses such as appointment adherence, which has not been investigated in previous work. Instead of looking at individual no-show probability using a logistic regression model, which is inappropriate as all appointments made by one patient cannot be viewed as independent samples, we proposed a coefficient comparison of two panel-DID models to elicit the difference in no-show rates attributable to the treatment effect. Our study provided scientific evidence of enhanced appointment adherence post portal activation. The same framework is also applicable to other type of care utilization investigations such as patients’ telephone encounters to primary clinics.

### Takeaways

Portal users were found to have fewer PCP office visits, adjusted by disease burden, compared to non-users in about one and a half year and thereafter post portal adoption. It suggests that the convenience brought by patient portal for supporting better provider-patient interaction might reduce patient in-person visit in a longer time-frame rather than immediately. This might possibly be explained as patients need time to adapt to portal functionalities, and patient portals influence patient health behavior gradually. In addition, it has been shown that patients with a high propensity to “no-show” for appointments will have worse clinical and acute care utilization outcomes compared to patients with a lower propensity [40]. We found that the no-show rates of portal users were lower compared to non-users, which suggests that portal access offers the promise to improve patient outcome. Moreover, missed appointments are known to critically impair clinic operational efficiency. In this study, the number of no-shows was estimated to be 0.14–0.20 per enrolled patient per year. Considering a PCP with a panel size of 1300 patients [41], the current no-show rate is equivalent to wasting an average of 182–260 appointment slots per provider. An average of 20% reduction, if it can be achieved through portal adoption, entails around forty less no-shows annually, or one less no-show weekly. Although not a significant improvement of provider productivity, the impact could be more pronounced by considering rescheduling no-show patients and other associated operating costs. On the other hand, it should be noted that portal access incurred an average of four messages sent from patients to their providers annually during the study period. If all 1300 panel patients turn into portal users, then a weekly average of 113 messages is expected for a single provider. Medical resources need to be allocated and physician appointment capacity needs to be adjusted to accommodate the demand shift introduced by increased patient portal adoption. Future work can be directed at investigating whether decreased PCP visits are associated with increased portal logins or messaging, and whether increased APN is associated with more portal logins or messaging. Additionally, payment structures that accommodate technologically-mediated interactions between providers and patients (e.g., text messaging, email, virtual visit, etc.) are instrumental in eliminating the adoption barrier of providers and should also be explored.

### Limitations

There are several limitations to our study. First, whether portal access affects health outcomes beyond care utilization needs to be systematically evaluated, and the business value and economic impact of portal usage need to be quantified. For the study design, the follow-up is limited to 10 quarters post adoption, and a longer observation horizon is beneficial to evaluate the long-run effect of portal adoption. Moreover, in addition to the overall portal effect, the usage of different portal features, such as appointment scheduling, lab results review, medication refill, and the messaging function, should be investigated. An analysis that incorporates the type and frequency of portal access by users can be informative. The user population was highly heterogeneous, and the impact of different portal features on subgroups can be diverse. Therefore, a subgroup analysis utilizing the detailed portal usage information should be carried out to investigate different portal features’ impact on patients. Lastly, the causal inference associating greater health concerns (e.g., number of chronic health conditions) and care utilization (e.g., number of office visits) cannot be established using the data collected for this study. Future research is needed to explore the causal factors of why patients seek and subsequently use portal services.

## Conclusions

The patient-centered care initiative heightened the awareness of health care systems’ responsibility to provide easily accessible ways for patients to be engaged in their own care by sharing the information necessary to empower patients to become effective health care partners. Understanding the impact of patient portal on patient engagement and care delivery efficiency is paramount to maximizing the potential of patient portal. Overall, our findings suggest that patient portal is effective in reducing no-show, but the relationship between portal adoption and primary care service utilization is more complex than the simple substitution of on-line for in-person care. It is vital to conceptualize the patient portal as a dynamic component of the patient-provider relationship and emphasize care coordination between patients and providers. Healthcare delivery planners and administrators should incorporate the impact of portal access into capacity planning and resource allocation, ultimately aligning patients’ and providers’ needs and functionality to enhance care delivery.

## Abbreviations

APN: Active problem number
DID: Difference-in-differences
EMR: Electronic medical record
PCP: Primary care physician
RR: Rate ratio
UF: University of Florida
